# Draft genome sequence of *bla*_IMP-1_-carrying *Phytobacter ursingii* OUH-01, isolated from the feces of an acute lymphoblastic leukemia patient

**DOI:** 10.1128/mra.00876-25

**Published:** 2026-03-12

**Authors:** Shuma Tsuji, Hideharu Hagiya, Shinnosuke Fukushima, Iori Urakami, Kazuyoshi Gotoh

**Affiliations:** 1 Department of Medical Laboratory Science, Okayama University Graduate School of Health Sciences, Okayama, Japan; 2 Department of Infectious Diseases, Okayama University Hospital, Okayama, Japan; 3 Research Center for Intestinal Health Science, Okayama University, Okayama, Japan; 4 Department of Bacteriology, Okayama University Graduate School of Medicine, Dentistry and Pharmaceutical Sciences, Okayama, Japan; Loyola University Chicago 2456, Chicago, Illinois, USA

**Keywords:** Enterobacteriaceae, carbapenems

## Abstract

We report the 5.7 Mbp draft genome of carbapenem-resistant *Phytobacter ursingii* strain OUH-01, recovered from the feces of a patient with acute lymphoblastic leukemia. The genome comprises a chromosome (G+C content of 53.5%), three plasmids, and four linear contigs, including the 222 kb plasmid pOUH-phyto-IMP-01 carrying *bla*_IMP-1_. Nanopore/Illumina sequencing achieved 235× coverage.

## ANNOUNCEMENT

*Phytobacter ursingii* strain OUH-01 was isolated in 2023 from a stool specimen collected during work-up of febrile neutropenia in an acute lymphoblastic leukemia patient at Okayama University Hospital, Japan. The clinical isolate was anonymized, and its use was deemed not to constitute human subjects research by the Okayama University IRB. CHROMagar mSuperCARBA (37°C, 24 h) yielded colonies suggestive of carbapenem-resistant *Enterobacterales* (CRE) whose meropenem MICs exceeded 16 µg/mL by broth microdilution (CLSI M100) (1). The modified carbapenem inactivation method was positive, and *bla*_IMP_ was detected with both GeneXpert Carba-R (Cepheid) and the Kaneka Carbapenemase Gene Detection Kit (Kaneka). MALDI Biotyper (Bruker Daltonics) identified the isolate as *P. ursingii*. Previous reports of acquired class C carbapenemase genes in *Phytobacter* spp. remain exceedingly rare, with only a few cases described globally (2–4). Notably, one such isolate was initially reported as *Metakosakonia massiliensis*, a species later reclassified into the genus *Phytobacter* (5). Therefore, we sequenced the whole genome of OUH-01.

Genomic DNA was purified from brain–heart-infusion agar cultures (37°C, 18 h) by lysozyme/achromopeptidase lysis, followed by phenol–chloroform extraction, as modified from Moria et al. (6). A ligation library prepared with SQK-NBD114-24 (Oxford Nanopore Technologies) was sequenced on a PromethION FLO-PRO114M flow cell, and reads were base-called using Dorado v7.2.14, yielding 609,870 reads (93.36 Gbp). An Illumina library constructed with the Illumina DNA Prep^(M)^ Tagmentation Kit (Illumina) was run on a NovaSeq6000 (2 × 151 bp), producing 2,498,489 paired reads (0.74 Gbp) that were quality-trimmed with fastp v0.20.1 (7). Hybrid assembly with Unicycler v0.4.8 (8) generated four circular contigs; polishing was used with Pilon v1.24 (9) twice because of no further base corrections. Circular contigs were automatically rotated to start near the cumulative GC skew minimum, which did not coincide with the *dnaA* locus. Annotation used DFAST v1.6.0 with the standard bundled reference database (“full” mode) (https://dfast.ddbj.nig.ac.jp/). PlasmidFinder v2.1 (10) classified the 222.9 kbp plasmid as Inc-type untypable. All software tools were run with default parameters unless otherwise specified.

The circulated contigs total 5,998,041 bp and consist of a 5,697,787 bp chromosome (G+C, 53.5%) plus three plasmids: pOUH-phyto-24k (23,841 bp; G+C, 61.0%), pOUH-phyto-54k (54,506 bp; 51.5%), and pOUH-phyto-IMP-01 (221,907 bp; 53.0%). The other four contigs were not closed as a circle. The chromosome encodes 5,813 coding sequences, 22 rRNA genes (8 × 5S, 7 × 16S, 7 × 23S), and 85 tRNAs. Hybrid sequencing yielded an average coverage of 235×.

Plasmid pOUH-phyto-IMP-01 carries *bla*_IMP-1_ embedded in a class 1 integron (*sul1-qacEΔ1*-attC-*bla*_IMP-1_-attI1-*intI1*) identified using IntegronFinder v2.0.6 (11), a structure commonly associated with transposition events. BLASTn revealed 99.9% identity over 220 kb to the Inc-untypable plasmid p2020-O-9 (GenBank acc. no. AP024848.1) of *Serratia marcescens* (12); however, *bla*_GES-5_ is replaced by *bla*_IMP-1_ in OUH-01, underscoring the mobility of this integron platform. The dDDH value (70.2%, 95% CI = 67.2%–73.1%) using GGDC v3.0 (13) against *P. ursingii* CAV1151 genomic sequence (GenBank acc. no. GCA_001022135.1) was at the species delineation threshold but, together with the GTDB (release R214) (14) search result of ANI = 96.14% and ΔG+C = 0.82%, supports classification as *P. ursingii*.

These data broaden the repertoire of IMP-positive *Phytobacter* genomes and highlight environmental *Enterobacterales* as hidden reservoirs of plasmid-borne carbapenemase genes.

## Data Availability

This whole-genome shotgun project has been deposited in DDBJ/ENA/GenBank under BioProject accession number PRJNA1268186 and assembly accession number GCA_050995525.1. Raw sequence reads from both Oxford Nanopore Technologies and Illumina platforms have been submitted to the NCBI Sequence Read Archive (SRA) under accession numbers SRR34861223 and SRR34861222. Genome annotation was performed using DFAST and is consistent with the sequence record.
